# Treatment of central nervous system infections caused by *Pseudomonas aeruginosa* with difficult-to-treat resistance: a narrative review

**DOI:** 10.3389/fmed.2026.1885309

**Published:** 2026-08-03

**Authors:** Chun Tao, Qiaozhi Hu

**Affiliations:** 1 Department of Pharmacy, Zigong Fourth People's Hospital, Zigong, China; 2 Department of Pharmacy, West China Hospital, Sichuan University, Chengdu, China

**Keywords:** blood-brain barrier, central nervous system infection, novel antimicrobial agents, *Pseudomonas aeruginosa* with difficult-to-treat resistance, therapeutic regimen

## Abstract

*Pseudomonas aeruginosa* with difficult-to-treat resistance (DTR-PA) refers to *Pseudomonas aeruginosa* that is non-susceptible to all first-line antimicrobial agents. With limited therapeutic options available, it poses a severe challenge to anti-infective therapy. For central nervous system infections caused by DTR-PA (DTR-PA-CNSI), the selection of antimicrobial agents is further restricted due to the presence of the blood-brain barrier. The continuous emergence of novel antimicrobial agents, especially new β-lactam antimicrobials, has provided more therapeutic alternatives for DTR-PA infections. This paper mainly discusses the pharmacokinetic/pharmacodynamic studies and clinical research of traditional antimicrobial agents (ceftolozane-tazobactam, ceftazidime-avibactam, aztreonam-avibactam, and cefiderocol) and novel antimicrobial agents (aminoglycosides, polymyxins, and fosfomycin) in the treatment of DTR-PA-CNSI, to evaluate their feasibility and the selection of therapeutic regimens for DTR-PA-CNSI and provide new strategies for clinical medication.

## 1 Introduction

*Pseudomonas aeruginosa* (PA) is an opportunistic pathogen that infects individuals with impaired host defense mechanisms in physiology, phagocytosis, or immunity, including patients with cystic fibrosis, non-cystic fibrosis bronchiectasis, hematological malignancies, and burns, and is associated with a high risk of mortality in these populations (1, 2). This pathogen readily adheres to medical devices such as indwelling catheters, ventilator circuits and implanted materials, where it exploits surface nutrients and local microenvironments to form mature biofilms; accordingly, PA ranks among the leading pathogens triggering device-associated biofilm infections (3). Furthermore, some clinical isolates of PA have been observed to compromise the structural integrity of medical devices, thereby raising the risk of device failure. Concurrently, this process provides a richer carbon source for the bacteria, thus promoting their growth and colonization (4). Once a medical device is contaminated, PA can invade the patient's body and cause infection. According to data from the National Healthcare Safety Network, PA is the fifth most common cause of healthcare-associated infections, ranking second, third, and tenth

in common causes of ventilator-associated pneumonia, catheter-associated urinary tract infection, and catheter-related bloodstream infection, respectively (5). PA is also one of the common pathogens causing central nervous system infection (CNSI) after neurosurgery. A meta-analysis of post-neurosurgical CNSI showed that PA accounted for more than 31% of cerebrospinal fluid (CSF) culture-positive samples (6), with a mortality rate ranging from 21% to 40% (7).

Carbapenem-resistant PA (CRPA) is defined as PA resistant to at least one carbapenem (imipenem, meropenem, etc.). A prospective cohort study demonstrated that 30-day mortality varied by CRPA infection site: 30% for bloodstream infection and only 7% for urinary tract infection (*p* = 0.0012) (8). PA with difficult-to-treat resistance (DTR-PA) is a specific category of CRPA, defined as PA non-susceptible to all first-line agents (piperacillin-tazobactam, aztreonam, ceftazidime, cefepime, imipenem-cilastatin, meropenem, ciprofloxacin, and levofloxacin) (9). Compared with CRPA, DTR-PA further highlights the limitations of antimicrobial options, and its definition is more consistent with clinical practice. Infections caused by DTR-PA have limited available agents, forcing clinicians to choose “rescue” drugs with high toxicity and low efficacy, leading to a high incidence of adverse drug reactions (ADRs) and a mortality rate of up to 50% (8, 10). Treatment of DTR-PA-induced CNSIs (DTR-PA-CNSIs) faces extraordinary obstacles owing to the blood-brain barrier (BBB), which demands antimicrobials to achieve sufficient therapeutic concentrations in CSF and further narrows eligible drug candidates. In recent years, with the launch of novel β-lactam antimicrobials and increasing research on DTR-PA-CNSI, more therapeutic options have become available for this condition (11). Therefore, this paper specifically focuses on DTR-PA-CNSI, reviews the mechanisms of DTR-PA resistance and risk factors for PA-CNSI, and integrates pharmacokinetic/pharmacodynamic (PK/PD) and clinical data on both traditional and novel antimicrobial agents to provide a tailored treatment regimen for DTR-PA-CNSI. It aims to address the shortcomings of existing reviews (12)—which tend to be overly generalized and lack species-specific analysis—and to serve as a reference for clinical practice.

## 2 Literature search

We adopted a structured narrative-review search strategy in accordance with the SANRA reporting standards, without rigorous procedures required for formal systematic reviews. We searched the PubMed, Web of Science, and Embase databases for literature on DTR-PA-CNSI treatment published between January 1, 2000, and April 30, 2026. The search keywords were as follows: *Pseudomonas aeruginosa*, carbapenem-resistant *Pseudomonas aeruginosa, Pseudomonas aeruginosa* with difficult-to-treat resistance, cefiderocol, ceftazidime–avibactam, imipenem–cilastatin–relebactam, aztreonam–avibactam, central nervous system infection, intracranial infection, meningitis, ventriculitis, et al. A combination of medical subject headings and free-text terms was applied in the retrieval strategy. Eligible study designs consisted of randomized controlled trials, case series and case reports, systematic reviews and meta-analyses, cohort studies, case-control studies, and clinical guidelines.

## 3 Resistance mechanisms of DTR-PA

PA harbors diverse antimicrobial resistance mechanisms (13–24), detailed as follows:

(1) Loss of outer membrane porins (e.g., OprD) and overexpression of efflux pumps, mediating resistance to β-lactams, fluoroquinolones, and aminoglycosides.
(2) Production of β-lactamases and other enzymes: cephalosporinases mediate resistance to penicillins and cephalosporins; carbapenemases (e.g., KPC-2, VIM) mediate resistance to carbapenems; aminoglycoside-modifying enzymes mediate resistance to aminoglycosides.
(3) Alteration of penicillin-binding protein targets, mediating resistance to β-lactams.
(4) Adaptive resistance: for instance, under antibiotic pressure, high bacterial density, low oxygen tension, and nutrient deprivation, a small subpopulation of PA enters a dormant or low-metabolic state to temporarily evade killing, forming “persister cells” that resume growth after removal of antibiotics.
(5) With regard to drug resistance, biofilm formation is a major factor, it is a type of adaptive resistance mechanism. The formation of biofilms enables PA to adhere firmly to various surfaces, such as catheters and ventilators, indicating the presence of chronic infections and long-term persistence. Under adverse conditions, after transitioning from a motile to a non-motile state, PA forms bacterial colonies. These colonies embed themselves in the extracellular matrix to protect themselves from the surrounding environment, forming what is known as a biofilm. The biofilm confers resistance to antibiotics, disinfectants, and host defense mechanisms, and the motile-non-motile transition increases the bacteria's antimicrobial resistance by hundreds of times.
(6) Other mechanisms: for instance, mutations in cell differentiation-related genes mediate resistance to ciprofloxacin and β-lactams.

The resistance of DTR-PA is generally mediated by the interaction of multiple mechanisms. PA secretes β-lactamases, alongside overexpression of efflux pumps and/or reduced outer membrane permeability caused by deletion or mutation of outer membrane porins. β-lactamases render antimicrobials inactive via hydrolysis of their β-lactam ring structure; efflux pumps actively pump antibiotics out of bacterial cells, whereas deletion or mutation of outer membrane porins restricts antimicrobial influx. Both pathways reduce intracellular drug concentrations (13, 16, 17, 20, 21, 25, 26). Their synergistic effect drastically elevates resistance. Clinically, monotherapy with β-lactamase inhibitors often fails to adequately counteract resistance; combined administration with efflux pump inhibitors or selection of antibiotics unaffected by both resistance pathways is essential. Mutations in penicillin-binding proteins weaken the binding capacity of antibiotics to their molecular targets. Meanwhile, β-lactamases degrade antibiotics and further cut down the level of active drugs (13, 25, 26). Synergy between these two mechanisms intensifies resistance, which requires the use of antibiotics insensitive to penicillin-binding proteins mutations or combination treatment regimens. Biofilm formation blocks antibiotic diffusion and prevents drugs from contacting bacterial cells. In addition, bacteria residing inside biofilms can further boost resistance through β-lactamase production, efflux pump overexpression and other pathways (13, 22, 24–26). Synergistic interplay of these resistance mechanisms greatly decreases the susceptibility of biofilm-embedded bacteria to antimicrobials and complicates treatment. For biofilm-associated infections, β-lactam monotherapy frequently delivers unsatisfactory outcomes. Therapeutic options include combination with biofilm-disrupting agents (e.g., azithromycin) or prescription of biofilm-penetrable antibiotics (e.g., cefiderocol) (13, 22, 24–26). In summary, multiple co-existent β-lactam resistance mechanisms in PA usually act synergistically and reinforce one another, leading to markedly heightened resistance. It is imperative that clinical management takes combined resistance profiles into full consideration in order to adopt targeted antibiotics or combination strategies, with a view to improving therapeutic efficacy and reducing the risk of treatment failure.

Data from the European Surveillance System (EARS-Net) in 2021 showed that 18.7% of PA isolates were resistant to fluoroquinolones, 18.7% to piperacillin-tazobactam, 18.1% to carbapenems, 15.8% to ceftazidime, and 8.9% to aminoglycosides (27). The prevalence of carbapenemase genes in CRPA isolates varied by region: highest in South and Central America (69%), 32% in China, and lowest in the United States (2%) (*p* = 0.0001) (8). Among 211 carbapenemase-producing isolates, KPC-2 (49%) and VIM-2 (36%) were the most common. Furthermore, CRPA isolates belong to distinct genetic lineages, with clone group CG235 being the most prevalent (12%), including 99% ST235 and 1% ST3746. CG235 is the dominant clone in South/Central America (27%), Australia and Singapore (23%), the Middle East (15%), and the US (9%). In South/Central America, 54% of CG111 isolates co-harbor bla_KPC − 2_ and bla_VIM − 2_ (8). In China, CG463 is the most common clone (20%), with 91% carrying bla_KPC − 2_, and it is not found in other regions. Most isolates in Australia and Singapore belong to CG308, mainly carrying bla_NDM − 1_ (8).

## 4 Risk factors for PA-CNSI

Several retrospective studies indicate that PA-CNSI in both adults and children is associated with neurosurgery (including drainage) (28–30). Other risk factors include oral and maxillofacial surgery, sinus surgery, CSF leakage, and leukemia (28–30). Bakhshi et al. retrospectively reviewed medical records of adult patients treated with lumbar drainage and found that CNSI was the most common complication, with PA as the predominant pathogen (31). Rodríguez et al. retrospectively analyzed 15 patients with PA-CNSI and found that 7 (46.6%) had a history of craniocerebral trauma (32).

PA is one of the common pathogens causing secondary bacterial infections in hospitalized patients with coronavirus disease 2019 (COVID-19). A meta-analysis of 30 studies including 3,834 patients showed that 7% of hospitalized COVID-19 patients developed bacterial infections, with PA as the second most common pathogen, accounting for 12% of secondary infections (33). COVID-19 is also considered a potential risk factor for PA-CNSI, as COVID-19 and its therapeutic agents induce immune dysregulation and increase the risk of CNSI by opportunistic pathogens including PA (34). Rhoades et al. found an increased abundance of PA in the nasal cavity of COVID-19 patients, positively correlated with viral RNA load (35). Furthermore, Mizuno et al. reported a term infant without immunosuppressive underlying diseases who developed PA-CNSI, and PA was also isolated from a household sponge used to clean feeding bottles, emphasizing the importance of environmental hygiene in reducing the incidence of PA-CNSI (36).

Regarding drug resistance, there are no specific studies on risk factors for drug-resistant PA-CNSI, but multiple studies have investigated risk factors for drug-resistant PA infection. As early as 2006, a multivariate model identified length of stay in the intensive care unit, bedridden status, invasive procedures, and prior use of broad-spectrum antimicrobials and aminoglycosides as significant risk factors for multidrug-resistant PA (MDR-PA) infection (37). For example, Corona et al. collected cases of post-external ventricular drain (EVD) CNSI, and 80.0% of PA isolates from CSF were carbapenem-resistant (29). Two recent retrospective studies evaluated risk factors for CRPA infection in children. One study of 172 children identified prior carbapenem use (OR: 0.102; 95% CI: 0.033–0.312; *P* < 0.001), bronchoscopy (OR: 0.147; 95% CI: 0.032–0.678; *P* = 0.014), invasive procedures in the past year (OR: 0.353; 95% CI: 0.159–0.780; *P* = 0.013), and β-lactam/β-lactamase inhibitor use in the past 90 days (OR: 0.327; 95% CI: 0.121–0.884; *P* = 0.03) as independent risk factors (38). Another study of 528 critically ill children with PA infection found that hospital stay >28 days (OR = 3.241, 95% CI 1.622–6.473, *p* = 0.001), invasive surgery (OR = 2.393, 95% CI 1.196–4.788, *p* = 0.014), and blood transfusion within 30 days before infection (OR = 7.003, 95% CI 2.416–20.297, *p* < 0.001) were significant risk factors for CRPA infection (39). Conversely, birth weight ≥2,500 g (OR = 0.278, 95% CI 0.122–0.635, *p* = 0.001) and breastfeeding (OR = 0.362, 95% CI 0.168–0.777, *p* = 0.009) were significant protective factors (39). In elderly inpatients, independent risk factors for CRPA infection included cerebrovascular disease (OR = 3.517, *P* < 0.001), indwelling urinary catheter (OR = 2.073, *P* = 0.018), length of hospital stay ≥14 days (OR = 1.980, *P* = 0.013), albumin < 35 g/L (OR = 2.049, *P* = 0.020), prior carbapenem use (OR = 7.022, *P* = 0.004), and prior third- or fourth-generation cephalosporin use (OR = 12.649, *P* = 0.002) (40).

## 5 Treatment strategies for DTR-PA-CNSI

According to the 2024 Infectious Diseases Society of America (IDSA) guidance for the treatment of antimicrobial-resistant Gram-negative infections (11), ceftolozane-tazobactam (C-T), ceftazidime-avibactam (CAZ-AVI), and imipenem-cilastatin-relebactam are preferred for DTR-PA infections, with cefiderocol as an alternative. In addition, the recently approved aztreonam-avibactam (ATM-AVI) is a potential first-line agent for DTR-PA infections (41–43). Traditional agents include aminoglycosides, polymyxins, and fosfomycin (11). Therefore, this article mainly discusses the research progress of the above medications in the treatment of DTR-PA-CNSI.

### 5.1 Traditional antimicrobials for DTR-PA-CNSI

#### 5.1.1 Aminoglycosides

Aminoglycosides are concentration-dependent bactericidal agents with low BBB penetration and a narrow therapeutic window, resulting in poor efficacy of intravenous administration for CNSI. They have long been administered via intraventricular/intrathecal (IVT/ITH) injection for CNSI caused by drug-resistant bacteria. For example, CSF concentrations of gentamicin are significantly above the susceptibility breakpoint after ITH administration of 4 mg (44). However, Gentamicin IVT/ITH doses exceeding 10 mg increase the risk of neurological ADRs and ototoxicity (45). Similarly, IVT administration of 8 mg amikacin achieves CSF concentrations of 150 μg/mL, and 5 mg achieves concentrations >100 μg/mL, both well above the susceptibility breakpoint ( ≤ 16 μg/mL) (46). A retrospective analysis of 51 PA-CNSI patients by Rodríguez et al. showed lower mortality in patients receiving ITH aminoglycosides (47).

The 2017 IDSA clinical practice guidelines for healthcare-associated ventriculitis and meningitis (48) recommend that the conventional adult IVT/ITH dosage of gentamicin is 4–8 mg, the pediatric dosage is 1–2 mg, and the standard dosage of amikacin is 30 mg, combined with IV therapy. Drainage tubes should be clamped for 0.5–2 h after local administration (Table 1). ADRs of local administration include transient hearing loss, seizures, aseptic meningitis, CSF eosinophilia, and painful radiculitis (only after ITH injection) (49, 50).

**Table 1:** Recommended antimicrobial agents and administration regimens for DTR-PA-CNSI.

Drug class	Recommended agent	Administration regimen	References	Comment	Evidence grade (GRADE)
Aminoglycosides	GTM	IVT(preferred)/ITH 4~8 mg, qd (adults), 1~2 mg, qd (children) + ivgtt	2017 IDSA	Clamp drain 0.5–2 h after local admin	Very low
	AMK	IVT (preferred)/ITH 5~50 mg (standard 30 mg), qd + ivgtt	2017 IDSA	Clamp drain 0.5–2 h after local admin	Very low
Polymyxins	PMB	IVT (preferred)/ITH 5 mg, qd + ivgtt	2017 IDSA	Clamp drain 0.5–2 h after local admin	Very low
	CMS	IVT (preferred)/ITH 10 mg, qd + ivgtt	2017 IDSA	Clamp drain 0.5–2 h after local admin	Very low
Fosfomycin	FOF	8g, q8h/6g, q6h, ivgtt >30 min/CI	Package insert; case reports	Limited evidence; Combine with other anti-PA agents	Very low
Novel β-lactams	C-T	3 g, q8h, ivgtt ≥ 3 h	2024 IDSA; case reports	Limited evidence; Combine with other anti-PA agents	Very low
	CAZ-AVI	2.5 g, q8h/q6h, ivgtt ≥ 3 h	2024 IDSA; case reports	Combine with other anti-PA agents; Optimized dosing improves PTA	Very low
	ATM-AVI	Loading 2.67 g, maintenance 2 g, q6h, ivgtt ≥ 3 h	Package insert	Limited evidence; Combine with other anti-PA agents	Very low
	CFD	2 g, q8h/q6h, ivgtt ≥ 3 h	2024 IDSA; case reports	Limited evidence; Combine with other anti-PA agents	Very low

GTM, gentamicin; IVT, intraventricular; ITH, intrathecal; qd, once daily; ivgtt, intravenous infusion; IDSA, Infectious Diseases Society of America; AMK, amikacin; PMB, polymyxin B sulfate; CMS, colistimethate sodium; FOF, fosfomycin; C-T, ceftolozane-tazobactam; q8h, every 8 h; CI, continuous infusion; PA, *Pseudomonas aeruginosa*; CAZ-AVI, ceftazidime-avibactam; q6h, every 6 h; PTA, probability of target attainment; ATM-AVI, aztreonam-avibactam; CFD, cefiderocol.

#### 5.1.2 Polymyxins

Polymyxins, mainly polymyxin B sulfate and colistimethate sodium (CMS), are concentration-dependent bactericidal agents active against drug-resistant Gram-negative bacilli but not Gram-positive cocci. Polymyxin B is inherently active and is primarily excreted via non-renal routes (only 4% via the kidneys) (51). CMS is a prodrug that requires conversion to active colistin and is mainly renally excreted (52). Polymyxins are hydrophilic drugs with a large relative molecular mass. Their BBB penetration rate is less than 5% after intravenous administration, making it difficult to achieve effective therapeutic concentrations in the CSF (53). In addition, due to their narrow therapeutic window, dose escalation will increase the incidence of ADRs such as nephrotoxicity (53). Therefore, local administration regimens are predominantly adopted for the treatment of CNSI. Multiple studies (54–56) show that IV plus IVT/ITH polymyxin B sulfate/CMS is the optimal regimen for MDR Gram-negative bacilli CNSI with high clinical cure rates. A retrospective study conducted by Liu et al. demonstrated that ITH administration of polymyxins serves as an effective therapeutic option for MDR-PA meningitis (28). Local administration of polymyxins can also reduce the mortality rate in patients with PA-CNSI (47).

The recommended IVT/ITH conventional dose is 50,000 U/day for polymyxin B and 10 mg/day for CMS, dissolved in ≥5 mL normal saline (48). Drainage tubes should be clamped for 0.5–2 h based on patient tolerance, CSF characteristics, and intracranial pressure. ADRs of IVT/ITH polymyxins include seizures, impaired consciousness, nuchal rigidity, fever, and muscle paralysis (57).

#### 5.1.3 Fosfomycin

Fosfomycin is a hydrophilic drug with a low relative molecular weight, and its BBB penetration can reach 50% under inflammatory conditions (58). Approximately 93%−99% of the drug is excreted unchanged in urine (59). Against PA, fosfomycin has a short post-antibiotic effect of 0.3–5.5 h. It exerts time-dependent or non-concentration-dependent bactericidal activity with a short half-life; hence, prolonged or continuous infusion achieves better PK/PD exposure (60, 61). It is available in oral and injectable formulations. The oral form is mainly used for lower urinary tract infections, while injectable fosfomycin sodium can be combined with other antimicrobials to treat susceptible bacterial infections at other sites, including CNSIs (62, 63). Albiero et al. (64) evaluated monotherapy and combination regimens of fosfomycin and meropenem against 19 clinical isolates of MDR-PA. The combination restored meropenem susceptibility in 40% of PA isolates (*n* = 10) (64). Compared with meropenem monotherapy alone, combination therapy with fosfomycin attained a probability of target attainment ≥90% in a larger proportion of tested strains (32% vs. 68%), demonstrating that fosfomycin delivers synergistic effects when used as a combination partner for PA infections.

The standard daily dose of injectable fosfomycin ranges from 12 to 24 g. For resistant bacterial pathogens, the recommended dosing regimens are 8 g every 8 h or 6 g every 6 h via intravenous drip or continuous infusion. Common ADRs include hypernatremia, hypokalemia, dysgeusia, hypersensitivity reactions, and phlebitis at the injection site (65–67).

### 5.2 Novel antimicrobials for DTR-PA-CNSI

#### 5.2.1 Ceftolozane-tazobactam

Ceftolozane is a novel antipseudomonal cephalosporin with high affinity for PA penicillin-binding proteins, 16 times more active against PA than ceftazidime (68). It is effective against PA with resistance mediated by efflux pump overexpression, OprD loss, and cephalosporinase overproduction, mainly used for drug-resistant PA infections including intra-abdominal infection, urinary tract infection, hospital-acquired bacterial pneumonia, and ventilator-associated bacterial pneumonia (68). Tazobactam inhibits β-lactamases (CTX-M, OXA, TEM, and SHV) in Enterobacterales to expand the spectrum, with efficacy like meropenem for pneumonia caused by ESBL-producing Enterobacterales (69). However, tazobactam does not enhance ceftolozane activity against PA, as PA-produced β-lactamases (OXA-2, PAmpC-3) do not hydrolyze ceftolozane, and tazobactam does not inhibit metallo-β-lactamases (MBLs), VIM, or KPC (70). Thus, tazobactam BBB penetration is irrelevant for DTR-PA-CNSI treatment. Ceftolozane (molecular weight 666.7) is hydrophilic with low plasma protein binding and is not a substrate for BBB transporters. In rats, brain tissue concentrations of 14C-labeled ceftolozane were below the limit of detection (12).

Clinical data on ceftolozane BBB penetration are conflicting and variable (9%−83%) (71–73). The largest study included 10 adult patients with ventricular drainage (5 with meningitis); after a single 3 g intravenous infusion (≥1 h), mean BBB penetration was 20%, higher in meningitis patients (35%) than non-meningitis (7%) (73). Standard-dose C-T achieves ≥90% probability of 40% fT > minimal inhibitory concentration (MIC) in CSF only when PA MIC ≤ 0.25 μg/mL, which may lead to suboptimal efficacy for Gram-negative meningitis unless MIC is low (73). This is based on single-dose data and may not reflect steady-state concentrations after multiple doses.

McCreary et al. reported a post-neurosurgical DTR-PA-CNSI patient cured with C-T (3 g q8h, ivgtt ≥1 h) + intravenous ciprofloxacin + IVT tobramycin, with significant improvement in intracranial infection and no recurrence at 1 year (71). Steady state ceftolozane CSF penetration was ~10%, achieving at least 50% fT>MIC. However, combination therapy was used, as with previously reported successful treatment cases (72, 74, 75), the contribution of C-T is unclear. Only 2 cases of C-T monotherapy for DTR-PA-CNSI have been reported: one achieved initial microbial clearance and clinical improvement but relapsed (76); another was cured with high-dose, extended-infusion C-T (77). In summary, despite limited PK/PD data and variable BBB penetration, multiple successful cases support C-T as a combination agent for DTR-PA-CNSI, especially when MIC ≤ 0.25 μg/mL (Table 1).

Common ADRs to C-T include allergic reactions (such as rash, fever, anaphylactic shock, etc.), gastrointestinal disturbances (such as nausea, vomiting, diarrhea, etc.), abnormal liver function, and bleeding (69, 78–80). Therefore, patients should be closely monitored for allergic reactions, gastrointestinal symptoms, liver function, coagulation function, and signs of bleeding during treatment.

#### 5.2.2 Ceftazidime-avibactam

CAZ-AVI is a β-lactam/β-lactamase inhibitor combination; avibactam inhibits class A, C, and some class D (OXA-48) β-lactamases, restoring ceftazidime activity (11). Avibactam (molecular weight 265.25) is water-soluble with low plasma protein binding and is a substrate for organic anion transporter 3. In rabbit meningitis models, BBB penetration of ceftazidime and avibactam was 43% and 38%, respectively, with significant reduction in CSF bacterial load (12). Case reports (30, 81–87) of CAZ-AVI for MDR-PA-CNSI are summarized in Table 2.

**Table 2:** Case reports of CAZ-AVI for multidrug-resistant PA-CNSI.

Study	Age (y)	Gender	Country	Comorbidity	Pathogen	Regimen	Duration	CSF culture conversion	ADR	Outcome
Yasmin et al. (81)	69	M	USA	subarachnoid hemorrhage	DTR-PA	CAZ-AVI 2.5 g, ivgtt, q8h + CMS 4.5 MIU, ivgtt, q12h + CMS 125000 IU, ITH q12h	≥21 d	Yes	None	Cured
Zhou et al. (82)	21	M	China	head trauma	DTR-PA	CAZ-AVI 1.25 g, ivgtt, q8h + AMK 600 mg, ivgtt, qd	20 d	Yes	None	Cured
Gofman et al. (83)	32	M	USA	cerebral hemorrhage	CRPA	CAZ-AVI 2.5 g, ivgtt, q8h + AMK 30 mg, ITH, qd	42 d	Yes	None	Cured
Almangourl et al. (84)	2	M	Saudi Arabia	hydrocephalus	DTR-PA	CAZ-AVI 187.5 mg/kg/d, ivgtt, q8h + CMS(D_1_: 30000 IU, IVT qd; D_2_-D_3_: 6000 IU, IVT qd; D_4_-D_14_: 125000 IU, IVT, qd)	21 d	Yes	None	Cured
Xipell et al. (85)	56	F	Spain	brain abscess	DTR-PA	CAZ-AVI 2.5 g, ivgtt, q8h + CMS 2 MIU, ivgtt, q8h	30 d	Yes	None	Cured
Yuan et al. (30)	14	M	China	ventricular tumor	DTR-PA	CAZ-AVI 2.5 g, ivgtt, q8h + CMS 125000 IU, ITH qd	14 d	Yes	None	Cured
Rodríguez-Núñez et al. (86)	58	F	Spain	sinusitis	XDR-PA	CAZ-AVI^*^, ivgtt + CMS^*^ ivgtt	38 d	Yes	None	NA
Gatti et al. (87)	52	M	Italy	pineal tumor	DTR-PA	CAZ-AVI 15 g, q8h, CI + FOF 24 g, qd, CI	24 d	Yes	None	Cured

DTR-PA, *Pseudomonas aeruginosa* with difficult-to-treat resistance; CAZ-AVI, ceftazidime-avibactam; ivgtt, intravenous infusion; q8h, every 8 h; q12h, every 12 h; qd, once daily; CMS, colistimethate sodium; ITH, intrathecal; AMK, amikacin; CRKP, carbapenem-resistant *Klebsiella pneumoniae*; CRPA, carbapenem-resistant *Pseudomonas aeruginosa*; IVT, intraventricular; XDR-PA, extensively drug-resistant *Pseudomonas aeruginosa*; ^*^, unspecified dosage; NA, not available; FOF, fosfomycin; CI, continuous infusion.

Xu et al. conducted a prospective observational study of CAZ-AVI for drug-resistant Gram-negative CNSI, showing 100% CSF target attainment, 71.4% microbial eradication, 100% clinical efficacy, and 85.7% clinical cure (*n* = 7) (88). Xipell et al. reported a DTR-PA-CNSI patient cured with IV CAZ-AVI + CMS. Clinically, CAZ-AVI is mainly combined with polymyxins or aminoglycosides (IV ± local) for DTR-PA-CNSI (85). Yasmin et al. cured a patient with standard-dose CAZ-AVI + IV/ITH CMS, with standard-dose CAZ-AVI achieving 50% fT≥MIC sufficient for bacterial killing (81). Gatti et al. reported that CAZ-AVI 2.5 g q6h increased fT>MIC to 100% without ADRs (87). CAZ-AVI also treats special CNSI, Yuan et al. cured a pediatric brain abscess with IV CAZ-AVI + ITH CMS. CAZ-AVI can also be combined with fosfomycin (30). A high-burden CNSI mouse model showed synergistic activity of CAZ-AVI + fosfomycin, significantly reducing PA load superior to monotherapy (89). Gatti et al. cured a DTR-PA-CNSI patient with CAZ-AVI + fosfomycin (87).

From the 8 reports in Table 2, the author further draws the following conclusions:

(1) Although additional evidence is still required for validation, combined with the above research findings, the author believes that CAZ-AVI can cross the BBB to achieve effective concentrations in CSF and thus be used for the treatment of DTR-PA-CNSI. At present, there are no efficacy evaluation results for monotherapy, and combination regimens are predominantly adopted. Common combinations include CMS (administered via intravenous and local routes), aminoglycosides (intravenous administration with or without local application), and fosfomycin (intravenous administration). The detailed dosing regimens are presented in Table 1.
(2) To date, no head-to-head studies on dual-drug combination therapy for DTR-PA-CNSI have been conducted. Further clinical research is therefore needed to verify its therapeutic efficacy and provide evidence for refined and individualized medication administration.
(3) Optimizing the administration strategy of CAZ-AVI, such as increasing the dosage and prolonging the infusion duration, can improve target attainment rates and enhance therapeutic outcomes.

Common ADRs to CAZ-AVI include allergic reactions (such as rash, itching, etc.), gastrointestinal symptoms (nausea, vomiting, abdominal pain, etc.), neurological ADRs (dizziness, headache, etc.), superinfections (such as vulvovaginal candidiasis and oral candidiasis, etc.), elevated transaminases, and hematological abnormalities (90–93). Therefore, patients should be monitored for allergic symptoms, gastrointestinal symptoms, neurological symptoms, signs and symptoms of vulvovaginal and oral infections, liver function, and complete blood count during treatment.

#### 5.2.3 Aztreonam-avibactam

ATM-AVI is a β-lactam/β-lactamase inhibitor combination; avibactam inhibits class A, C, and some class D β-lactamases, restoring aztreonam activity, ATM is not hydrolyzed by MBL (class B β-lactamases) (41–43). Therefore, compared to CAZ-AVI, ATM-AVI exhibits antimicrobial activity against MBL-producing strains and is primarily used clinically to treat infections caused by such strains. Consequently, it is advisable to perform an MBL test before administration to ensure targeted use of the drug. No PK/PD or clinical studies of ATM-AVI for DTR-PA-CNSI exist, but cases of CAZ-AVI + aztreonam for MBL-producing strains have been reported (94, 95). Madoure et al. reported a post-neurosurgical intracranial infection with MBL-producing *Klebsiella pneumoniae* controlled by CAZ-AVI + aztreonam + tigecycline after amikacin failure (94). Wong et al. cured a post-neurosurgical *Stenotrophomonas maltophilia* CNSI with CAZ-AVI (2.5 g q8h) + aztreonam (2 g q8h), with CSF concentrations above MIC at all time points (95). Prior confirmation of strain cross-susceptibility or drug synergistic effects is required when applying this two-drug regimen to treat PA infections, as multiple resistance mechanisms harbored by PA can directly compromise clinical efficacy.

At present, there are no relevant studies on the use of ATM-AVI for the treatment of DTR-PA-CNSI. However, both aztreonam and avibactam exhibit favorable BBB penetration, enabling them to reach effective therapeutic concentrations in CSF. In addition, successful clinical cases of CNSI treated with the combined regimen of CAZ-AVI plus ATM have been reported. Therefore, although further PK/PD studies and clinical trials are still required to validate its efficacy, the author suggests that ATM-AVI can serve as a potential therapeutic option for DTR-PA-CNSI, with the specific dosing regimens summarized in Table 1.

ATM-AVI was generally well tolerated, and safety findings were consistent with the known safety profile of aztreonam monotherapy (43). Common ADRs include anemia, thrombocytopenia or thrombocytosis, elevated transaminases, gastrointestinal adverse reactions (such as nausea, vomiting, diarrhea, and abdominal pain), altered mental status, and infusion-related adverse reactions (43, 96). Therefore, patients should be monitored for complete blood count, liver function, gastrointestinal symptoms, mental status, and infusion-related ADRs during treatment.

#### 5.2.4 Cefiderocol

Cefiderocol is a catechol-containing “siderophore cephalosporin” that binds extracellular iron and is actively transported into PA via iron transporters for bactericidal activity (97). In rat meningitis models, BBB penetration is like ceftriaxone and cefoperazone: 6% without inflammation, 15%−18% with inflammation (12). Cefiderocol has high susceptibility to PA (97.3%−99.8%) (98, 99) and potent *in vitro* activity (100). Clinical studies (100) show efficacy for MDR non-fermenter infections: superior to polymyxins for *Acinetobacter baumannii* (lower mortality, nephrotoxicity), reliable for *Stenotrophomonas maltophilia*, and durable for PA with low resistance induction. Case reports (101–104) of cefiderocol for MDR-PA-CNSI are summarized in Table 3.

**Table 3:** Case reports of cefiderocol for multidrug-resistant PA-CNSI.

Study	Age (y)	Gender	Country	Comorbidity	Pathogen	Regimen	Duration	CSF culture conversion	ADR	Outcome
Stevenson et al. (101)	41	F	UK	Cerebral hemorrhage	DTR-PA	CFD 1 g, ivgtt, q8h + CMS 5MIU, ivgtt, q12h CFD 1.5 g, ivgtt, q8h + CMS 6 MIU, ivgtt, q12h + CMS 0.125 MIU, ITH, qd	12 d	Yes	None	Death (non-infectious)
Marcelo et al. (102)	63	M	Spain	Meningioma	DTR-PA	CFD 2 g, ivgtt, q8h + CMS 4.5 MIU, ivgtt, q12h	32 d	Yes	None	Cured
Luque-Paz et al. (103)	71	NA	France	Craniopharyngioma	DTR-PA	CFD 2 g, ivgtt, q8h + CMS 10 mg, IVT, qd + AMK 30 mg, IVT, qd	15 d	No	None	Death
Sollima et al. (104)	60	M	Italy	Subarachnoid hemorrhage	MDR-PA	CFD 2 g, ivgtt, q8h + TBM^*^+ CFX^*^	22 d	Yes	None	Death (non-infectious)
Sollima et al. (104)	NA	M	Italy	Lung transplant	MDR-PA	CFD 2 g, ivgtt, q8h + FOF^*^	3 mon	Yes	None	Cured
Sollima et al. (104)	55	F	Italy	Hydrocephalus	DTR-PA	CFD 2 g, ivgtt, q8h + FOF^*^	NA	Yes	None	Cured
Sollima et al. (104)	74	M	Italy	Meningioma	XDR-PA	CFD^*^	21 d	Yes	None	Cured

DTR-PA, *Pseudomonas aeruginosa* with difficult-to-treat resistance; XDR-PA, extensively drug-resistant *Pseudomonas aeruginosa*; CFD, cefiderocol; q8h, every 8 h; q12h, every 12 h; qd, once daily; CMS, colistimethate sodium; IVT, intraventricular; ITH, intrathecal; AMK, amikacin; MDR-PA, multidrug-resistant *Pseudomonas aeruginosa*; TBM, tobramycin; CFX, ciprofloxacin; FOF, fosfomycin; ^*^, unspecified dosage; NA, not available.

No PK/PD studies of cefiderocol in the CNS have been conducted in drug registration trials, and relevant subsequent research remains extremely limited (105–107). Stevenson et al. administered a reduced dose of cefiderocol (1.5 g intravenously every 8 h) combined with CMS (intravenous plus ITH administration) to treat one patient with DTR-PA-CNSI, achieving microbiological cure (101). A total of five CSF samples were collected for drug concentration monitoring, and the results showed that CSF drug concentrations fluctuated around the MIC, with two measurements below the MIC and three above the MIC. Although current evidence from clinical studies is lacking, the author believes that the therapeutic efficacy was most likely attributed to the synergistic effect of the two agents. Moreover, administration of standard doses or individualized dose adjustment based on serum and CSF drug concentrations would enable cefiderocol to achieve more effective CSF concentrations, which is consistent with the findings reported by Luque-Paz et al. (103) and Meschiari et al. (108). The case report published by Kufel et al. further supports the application of cefiderocol in the treatment of CNSI (109). In their study, two standard dosing regimens (2 g intravenous infusion every 6 h and 2 g intravenous infusion every 8 h) were used to successfully cure a patient with *Acinetobacter baumannii*-induced CNSI. The estimated BBB penetration rates of the two regimens were 68% and 60%, respectively.

No head-to-head comparisons with other novel β-lactams exist, but cefiderocol is an excellent salvage agent for treatment failure/resistance to CAZ-AVI/C-T. Luque-Paz et al. (103) reported a DTR-PA-CNSI patient resistant to CAZ-AVI/C-T, cured with cefiderocol 2 g q8h + IV/ITH polymyxin + IVT amikacin, with CSF concentrations 80–100 × MIC. Colombo et al. reported a case of CNSI caused by Carbapenem-Resistant *Klebsiella pneumonia*e following neurosurgical intervention (110). The patient initially received anti-infective treatment with CAZ-AVI combined with fosfomycin and showed early clinical improvement, followed by subsequent disease deterioration. After the treatment was switched to cefiderocol (2 g administered intravenously four times daily), the patient was successfully cured. Sollima et al. (104) also reported similar salvage cases.

From the seven reports summarized in Table 3, the author further draws the following conclusions:

(1) Although further research is still required for evidence support, the author believes that cefiderocol can penetrate the BBB to achieve effective therapeutic concentrations in CSF and is applicable for the treatment of DTR-PA-CNSI. The corresponding dosing regimens are shown in Table 1;
(2) Cefiderocol is mainly administered in combination with other antimicrobial agents. The primary combination partner is CMS (intravenous administration plus IVT/ITH administration). It can also be combined with fosfomycin (intravenous administration) and aminoglycosides (intravenous administration with or without IVT/ITH administration);
(3) Optimizing the administration route and dosage can increase the CSF concentration of cefiderocol, thereby improving clinical efficacy.

Common ADRs to cefiderocol are allergic reactions, gastrointestinal ADRs (such as nausea, vomiting, diarrhea, constipation, etc.), neurological ADRs (such as headache, coma, tremor, myoclonus, seizures, etc.), oral ulcers, cough, liver dysfunction, infusion reactions, and swelling (107, 111, 112). Therefore, patients should be monitored for allergic symptoms, gastrointestinal symptoms, neurological symptoms, oral conditions, respiratory symptoms, liver function, and infusion-related ADRs during treatment.

#### 5.2.5 Imipenem-cilastatin-relebactam

Imipenem-cilastatin-relebactam is a β-lactam/β-lactamase inhibitor combination; relebactam inhibits class A and C β-lactamases, restoring imipenem activity, with good efficacy for imipenem-resistant PA (11). However, no BBB penetration studies in animals or clinical data for DTR-PA-CNSI exist. Imipenem has high BBB penetration but a higher seizure risk than other carbapenems, making it unsuitable for CNSI (113). Thus, imipenem-cilastatin-relebactam is **not recommended** for DTR-PA-CNSI.

Susceptibility of DTR-PA to novel β-lactams varies by region due to differing resistance mechanisms. US data (114, 115) show high susceptibility of CRPA to C-T (90%), CAZ-AVI (85%), imipenem-cilastatin-relebactam (86%), and cefiderocol (99%), as only 2% of CRPA produce carbapenemases (8). CAZ-AVI is inactive against MBL-producing PA (VIM, NDM), leading to higher resistance in Latin America and the Middle East (8, 116). C-T is inactive against KPC- and MBL-producing PA, leading to higher resistance in Latin America and China (8, 116). For DTR-PA-CNSI, susceptibility testing of all theoretically active novel β-lactams is recommended; carbapenemase typing is preferred in high-prevalence regions to guide therapy. Furthermore, access to new β-lactam antibiotics varies across different regions of the world. Overall, access to new antibiotics is closely related to regional economic levels, the sophistication of healthcare systems, policy support, and international cooperation. Accessibility is higher in high-income countries, while low- and middle-income countries—particularly in Africa and remote regions—face severe challenges (117, 118). Accessibility must be improved through measures such as international aid, local production, and price regulation (117, 118). Therefore, drug selection must also be tailored to local conditions.

The existing evidence relevant to this study has limitations. Currently, most clinical conclusions regarding specific drugs are derived from case reports and small-sample series studies, which have limited sample sizes and lack support from large-scale controlled trials. At the same time, such positive outcomes are more likely to be published in journals, creating a potential publication bias that may overestimate the actual efficacy and safety of the drugs.

## 6 Conclusion

DTR-PA-CNSI poses a major clinical challenge due to limited therapeutic options. Available data support the use of traditional agents (aminoglycosides, polymyxins, and fosfomycin) and novel β-lactams (C-T, CAZ-AVI, and cefiderocol) for treatment. Based on the good BBB permeability of its constituent components, as well as indirect analogy cases, we also recommend ATM-AVI for the treatment of DTR-PA-CNSI. Imipenem-cilastatin-relebactam is not recommended due to lack of CNS PK data and increased seizure risk. Traditional agents are irreplaceable due to low cost and abundant data, providing a foundation for combination therapy with novel agents, but have limitations including suboptimal efficacy, high ADRs risk, and technical complexity; individualized selection and close monitoring of efficacy and ADRs are required. Novel agents offer reliable efficacy and favorable safety profiles, expanding therapeutic options, but have limited CNS PK/PD and clinical data, suitable for combination therapy. In addition, regional accessibility of these novel antimicrobials should also be considered during therapeutic selection. Future research should focus on more clinical and PK/PD studies to evaluate feasibility and optimize dosing for safer and more effective DTR-PA-CNSI treatment. With the launch of more novel β-lactams and other classes, we anticipate more BBB-penetrating agents to improve outcomes for DTR-PA-CNSI.
